# Leaky resistance and the conditions for the existence of lytic bacteriophage

**DOI:** 10.1371/journal.pbio.2005971

**Published:** 2018-08-16

**Authors:** Waqas N. Chaudhry, Maroš Pleška, Nilang N. Shah, Howard Weiss, Ingrid C. McCall, Justin R. Meyer, Animesh Gupta, Călin C. Guet, Bruce R. Levin

**Affiliations:** 1 Department of Biology, Emory University, Atlanta, Georgia, United States of America; 2 Institute of Science and Technology Austria, Klosterneuburg, Austria; 3 School of Mathematics, Georgia Institute of Technology, Atlanta, Georgia, United States of America; 4 Division of Biological Sciences, University of California San Diego, La Jolla, California, United States of America; 5 Department of Physics, University of California San Diego, La Jolla, California, United States of America; Wageningen Universiteit en Researchcentrum, The Netherlands

## Abstract

In experimental cultures, when bacteria are mixed with lytic (virulent) bacteriophage, bacterial cells resistant to the phage commonly emerge and become the dominant population of bacteria. Following the ascent of resistant mutants, the densities of bacteria in these simple communities become limited by resources rather than the phage. Despite the evolution of resistant hosts, upon which the phage cannot replicate, the lytic phage population is most commonly maintained in an apparently stable state with the resistant bacteria. Several mechanisms have been put forward to account for this result. Here we report the results of population dynamic/evolution experiments with a virulent mutant of phage Lambda, λ^VIR^, and *Escherichia coli* in serial transfer cultures. We show that, following the ascent of λ^VIR^-resistant bacteria, λ^VIR^ is maintained in the majority of cases in maltose-limited minimal media and in all cases in nutrient-rich broth. Using mathematical models and experiments, we show that the dominant mechanism responsible for maintenance of λ^VIR^ in these resource-limited populations dominated by resistant *E*. *coli* is a high rate of either phenotypic or genetic transition from resistance to susceptibility—a hitherto undemonstrated mechanism we term "leaky resistance." We discuss the implications of leaky resistance to our understanding of the conditions for the maintenance of phage in populations of bacteria—their “existence conditions.”

## Author summary

While it is clear that bacteriophage abound in bacterial communities, their role in the ecology and evolution of these communities remains poorly understood. Fundamental questions remain unanswered, such as, are phage regulating the population densities of their host bacteria? And how are virulent phage maintained in bacterial communities, following the seemingly inevitable evolution of resistant bacteria? Here we present a theoretical and experimental investigation to provide evidence for a new mechanism for maintaining phage in populations dominated by resistant bacteria. This mechanism, which we term “leaky resistance,” is based on a high rate of either phenotypic or genetic transition from resistance to susceptibility.

## Introduction

The viruses of bacteria and archaea, phage, for brevity and generality, are touted to be the most abundant organisms on Earth [1]. Research with phage, and particularly but not exclusively those that infect *E*. *coli*, have played a major role in the development of the contemporary concept of the gene [2], the demonstration that DNA is the genetic material [3], "breaking" the genetic code [4], understanding the regulation of gene action [5], and the development of classical (restriction-endonuclease-based) [6] and contemporary CRISPR-Cas (clustered regularly interspaced short palindromic repeats, CRISPR-associated proteins)–based genetic engineering [7]. Phage have been used to prevent and treat bacterial infections [8], an enterprise that, in response to concerns about mounting resistance to antibiotics, is being resurrected in various ways [9–12]. Phage are also significant as a source of contamination for the bacteria employed in processing of dairy products [13,14], as well as other industrial applications [15].

Despite the abundance and ubiquity of phage in natural bacterial and archaeal communities, including the enteric microbiomes of humans [16,17], together with all that we know about their structure, genetics, molecular biology, and mechanisms of replication, relatively little is known about the ecology and ecological role of phage in bacterial communities. What are the ecological, genetic, and evolutionary conditions necessary for phage to be maintained in bacterial populations? In the words of Allan Campbell, what are their "existence conditions" [18]? Do phage regulate the densities of their host populations of bacteria and archaea? What is the role of phage in determining the distribution and abundance of different species and genotypes of bacteria in their natural communities?

While the answers to these questions may be unknown for natural populations, these questions have been addressed with mathematical models, as well as experiments with bacteria and lytic (“virulent”) phage in continuous and serial passage cultures. In theory, if the densities of susceptible host populations are sufficiently high, lytic phage can become established and regulate the densities of these populations under a broad set of conditions [18–20], and this has been observed experimentally [21,22]. However, as a consequence of mutation to resistance or, in the case of CRISPR-Cas, acquired immunity, bacteria upon which the lytic phage cannot replicate eventually emerge, and these experimental bacterial populations become limited by resources rather than phage [22–30]. While the lytic phage may eventually be lost in such cultures [26,31], in most cases they are maintained and coexist with bacteria resistant or immune to them for extensive periods of time [32].

Several mechanisms have been proposed to account for how lytic phage are maintained in populations dominated by bacteria upon which they cannot replicate: (i) the resistant bacteria are at a strong selective disadvantage. As a result of this disadvantage, a minority population of phage-susceptible bacteria continues to be maintained in a population dominated by resistant cells, and supports replication of the phage [20,22,33,34]. (ii) There is an indefinite coevolutionary arms race between the bacteria and the phage. The emergence and ascent of bacterial resistance to phage selects for host range phage mutants that can grow on the resistant bacteria. This, in turn, selects for new mutations that generate bacteria resistant to the newly evolved phage and so on [26,35]. (iii) The habitat is heterogeneous. A small population of susceptible bacteria (refuge) is maintained in a subhabitat that is inaccessible to the phage and continues to provide susceptible cells in cultures dominated by resistant bacteria [36].

In addition to these mechanisms, a hypothesis was presented by Max Delbrück more than 70 years ago [37] to account for how, in cultures of resistant bacteria “contaminated with virus,” the phage continued to be maintained. He postulated that susceptible cells were continually “thrown off” by mutation and, by replicating on these susceptible cells, the phage are able to maintain their population in a community dominated by bacteria upon which they cannot replicate. Delbruck did not explore in the necessarily quantitative way the conditions under which this mechanism could account for the maintenance of the phage or test this hypothesis experimentally. It was, however, clear that he found this mechanism more appealing than lysogeny, which could have also explained the continued presence of phage in a population of seemingly resistant cells. Far more recently, Weissman and colleagues explored the conditions under which this mechanism can account for the continued maintenance of phage in populations dominated by immune bacteria carrying CRISPR-Cas [31]. General theoretical consideration and direct experimental verification of this hypothesis are, however, still lacking.

The results of our computer simulations and experiments using *E*. *coli* and a virulent mutant of phage λ (λ^VIR^) provide compelling evidence in support of the hypothesis that high rates of genetic and/or phenotypic reversion from resistance to susceptibility, a phenomenon we term “leaky resistance,” is the dominant if not the unique mechanism responsible for continued maintenance of λ^VIR^ in populations dominated by λ^VIR^-resistant *E*. *coli*.

## Results

### Theoretical considerations

To generate hypotheses and to facilitate the design and interpretation of the results of experiments, we use a simple, mass action mathematical model of population and evolutionary dynamics of bacteria and phage in a liquid culture. This model is a version of that described in [19] modified for serial transfer, rather than a continuous (chemostat) culture. We consider two populations of bacteria: a susceptible population *N* (cells per mL), upon which the phage can replicate, and a resistant population *N*_*R*_ (cells per mL), to which the phage does not adsorb. As in [38,39], we assume that the bacteria grow at rates equal to the product of their maximum growth rates, *v* and *v*_*R*_ (per cell per hour), for susceptible and resistant bacteria, respectively, and a Monod function: *ψ*(*R*) = *R*/(*k* + *R*), where *R* is the concentration (μg per mL) of the limiting resource and *k* is the “Monod constant,” corresponding to the concentration of the resource (μg per mL), at which the bacteria grow at a half of their maximum rate. As in [39], we assume that the bacteria take up the resource at a rate jointly proportional to their density, their growth rate, and a conversion efficacy parameter, *e* (μg per cell).

Lytic phage, which are present at density *V* (particles per mL), adsorb to the susceptible bacteria according to the law of mass action at a rate equal to the product of their densities and a rate constant *δ* (per hour per mL) [19]. For simplicity, we disregard the latent period and assume that the infected bacteria are killed instantaneously, liberating *β*-1 phage particles (the burst size minus the one phage particle lost by adsorption). At rates *μ*_*N*_ and *μ*_*R*_ (per cell per hour), respectively, susceptible bacteria become resistant to the phage (*N*_*S*_→*N*_*R*_ transition) and resistant bacteria become susceptible (*N*_*R*_→*N*_*S*_ transition). To account for the decline in the rates of metabolism as bacteria approach stationary phase, we assume that the rates of transition as well as the rate at which the phage adsorb to the bacteria decline with the concentration of the limiting resource by multiplying the rates parameters by *ψ(R)*. With these definitions and assumptions, the rates of change in concentration of the resource and densities of bacteria and phage are given by a set of coupled differential equations:

$$\frac{dR}{dt}\;=\;\underset{resource\;uptake}{\underbrace{-e\cdot \psi (R)\cdot (v\cdot N+v_{r}\cdot N_{r})}}$$ (1)

$$\frac{dN}{dt}\;=\;\underset{growth}{\underbrace{v\cdot \psi (R)\cdot N}}-\underset{phage\;lysis}{\underbrace{\delta \cdot \psi (R)\cdot N\cdot V}}+\underset{transition}{\underbrace{\left(\mu_{R}\cdot N_{R}-\mu_{N}\cdot N)\cdot \psi (R\right)}}$$ (2)

$$\frac{dN_{R}}{dt}\;=\;\underset{growth}{\underbrace{v_{R}\cdot \psi (R)\cdot N_{R}}}+\underset{transition}{\underbrace{\left(\mu_{N}\cdot N-\mu_{R}\cdot N_{R})\cdot \psi (R\right)}}$$ (3)

$$\frac{dV}{dt}\;=\;\underset{phage\;lysis}{\underbrace{\delta \cdot \psi (R)\cdot N\cdot V\cdot (\beta -1)}}$$ (4)

where *ψ*(*R*) = *R*/(*k* + *R*).

In our simulations, we assume the existence of a refuge density *ref* (cells per mL) for the susceptible bacteria, perhaps wall populations [36], below which the phage cannot adsorb to them. To simulate the serial transfer protocol used in our experiments, the density of the bacteria and phage populations as well as the concentration of the resource are reduced every 24 hours by a factor of *d* = 0.01. At the same time, a defined amount *C* (μg per mL) of fresh resource is added. For convenience, we assume a culture volume of 1 mL.

**Fig 1:**
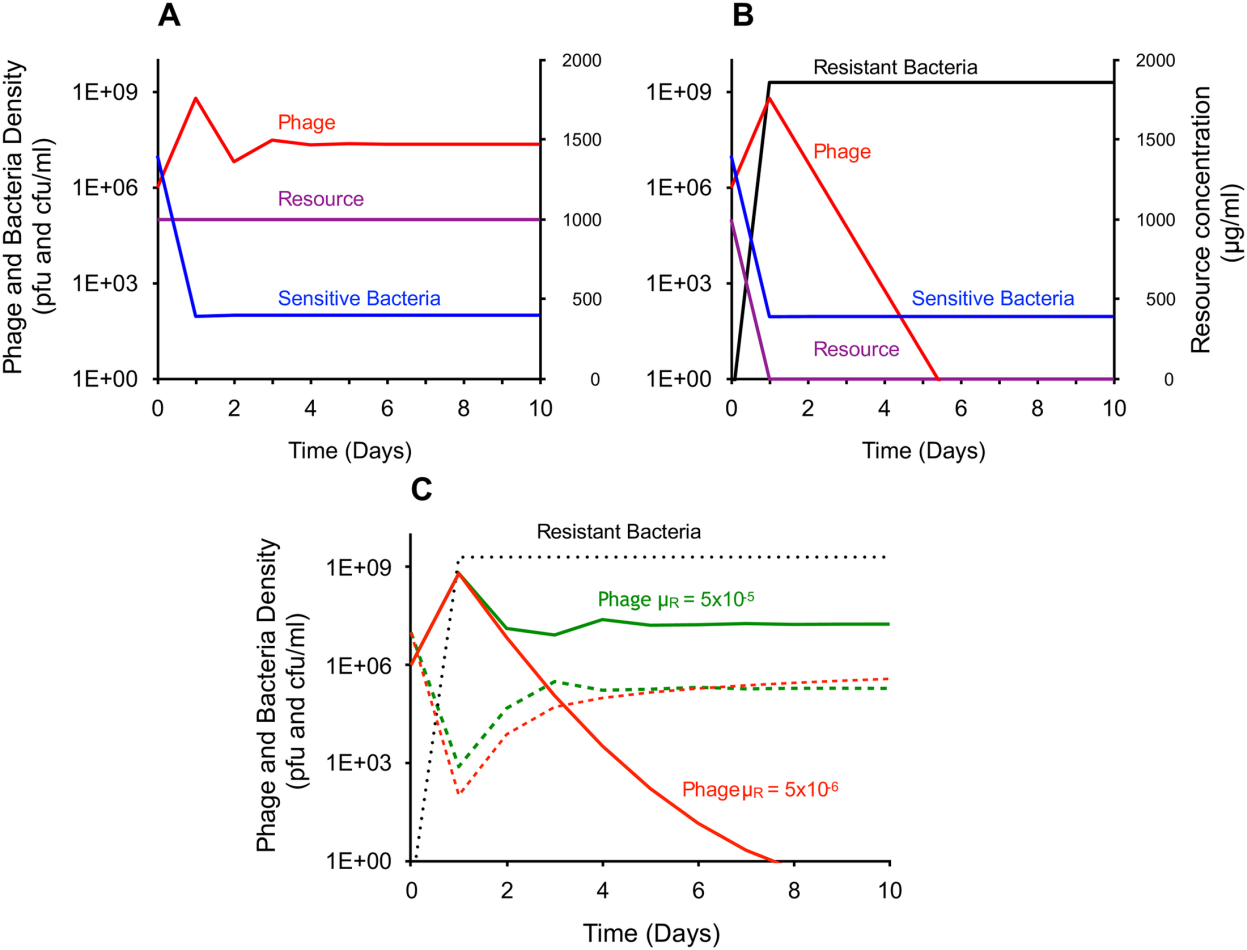
Simulations of population dynamics of bacteria and lytic phage in serial transfer culture. **(A)** Serial transfer dynamics assuming no transition between susceptible and resistant states (*μ*_*N*_ = *μ*_*R*_ = 0) with a refuge of *ref* = 10^2^. **(B)** Serial transfer populations considering transition from susceptibility to resistance (*μ*_*N*_ = 5 × 10^−6^). *μ*_*N*_ was estimated independently. Transition from susceptibility to resistance is not allowed (*μ*_*R*_ = 0). ref = 10^2^. **(C)** Serial transfer populations considering both transition from susceptibility to resistance and vice versa. Solid curves represent phage densities. Dashed curves represent densities of susceptible bacteria. The black dotted line represents density of resistant bacteria for both simulations. Resource concentration is not shown. In all simulations, densities are plotted at the end of each transfer. Parameters used: *v* = *v*_*r*_ = 1, *k* = 1, *δ* = 2 × 10^7^, *β* = 60, *C* = 1,000, *e* = 5 × 10^7^. The parameters were estimated independently in LB medium. The simulations were initiated with 10^7^ susceptible bacteria and 10^6^ phage. The models were solved numerically using the Berkeley Madonna software. Copies of this and the other program used in this study are available at www.eclf.net. Underlying data can be found in S1 Data. cfu, colony-forming unit; LB, Lysogeny broth; pfu, plaque-forming unit.

In Fig 1 we present the results of numerical simulations with the parameters in the range estimated for Lysogeny broth (LB) medium. In the absence of transition from susceptibility to resistance (*μ*_*N*_ = 0), the phage density rapidly increases and that of the susceptible bacteria declines to the refuge level (Fig 1A). In the course of a serial transfer, in which one hundredth of the culture is transferred to fresh medium every 24 hours, the bacteria continue to be maintained at the refuge level, with the concentration of the resource being only slightly lower than the initial concentration at each transfer. Thus, the density of the bacterial population is limited by the phage. A very different result obtains if we allow for transition from susceptibility to resistance (*μ*_*N*_ = 5 × 10^−6^), without yet considering transition in the opposite direction (*μ*_*R*_ = 0). A population of bacteria resistant to the phage is generated and ascends, whilst that of the susceptible bacteria declines (Fig 1B). The concentration of the resource at the end of each transfer approaches zero, indicating that the resource, rather than phage, limits the bacterial population. Despite that susceptible bacteria are present, the rate of phage infection is too low for the phage to maintain their population. In other words, the phage are lost because the extent to which they are produced in each transfer is lower than the rate required to overcome the serial 100-fold dilutions.

Once transition from resistance to susceptibility is allowed, the phage can be stably maintained in a population dominated by resistant bacteria because of the existence of a sufficiently large subpopulation of susceptible cells (Fig 1C). However, to achieve stable phage maintenance, the transition rate from resistance to susceptibility has to be noticeably large (at the order of 10^−5^ per cell per hour or greater). Below this rate of transition from resistance to susceptibility, the density of susceptible bacteria is too low for the phage to stably maintain its population. It deserves to be noted that because of inaccuracies in estimating the parameters and the many simplifying assumptions of the model, the value of the threshold transition rate above which the phage can be maintained is, at best, approximate.

### Experimental results

#### Population dynamics of λ^VIR^ and *E*. *coli* in serial transfer culture

**Fig 2:**
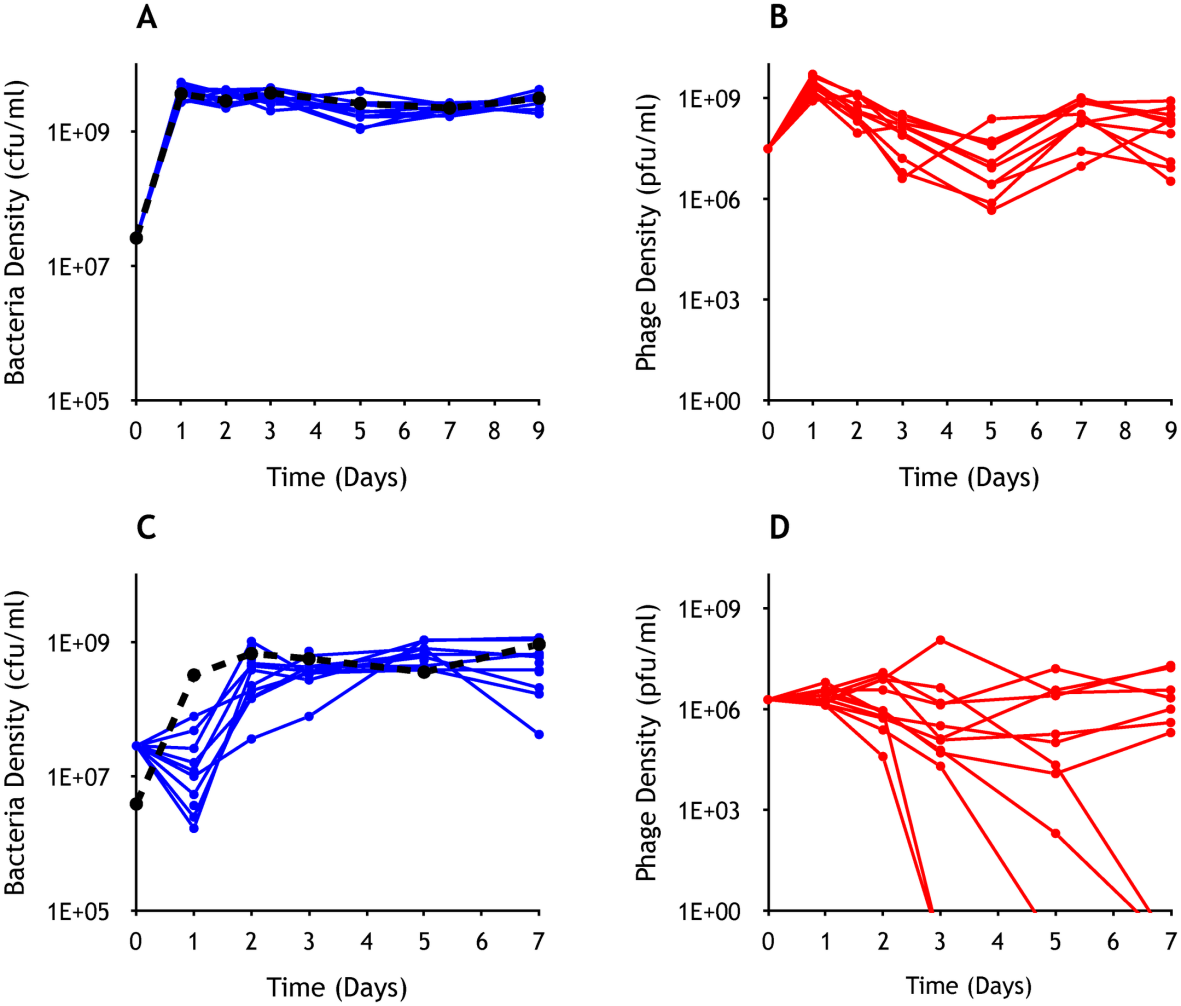
Experimentally determined population dynamics of bacteria and lytic phage in serial transfer culture. **(A)** Changes in bacterial densities in 10 LB cultures. **(B)** Changes in phage densities in 10 LB cultures. **(C)** Changes in bacterial densities in 12 M9M cultures. **(D)** Changes in phage densities in 12 M9M cultures. The control populations (black dashed curves) represent bacterial densities in cultures grown in the absence of λ^VIR^. In LB cultures (A and B), the densities of bacteria and phage were estimated at the end the first three transfers and then every second transfer. In M9M cultures (C and D), the density of bacteria and phage was estimated at the end of each transfer. The medium used had a statistically significant effect on phage maintenance (*p* = 0.002, with 22 observations at 20 degrees of freedom), as calculated using logistic regression, with the medium used (LB/M9M) as a categorical independent variable and phage maintenance (maintained/lost) as a binary dependent variable. Underlying data can be found in S1 Data. cfu, colony-forming unit; LB, Lysogeny broth; M9M, M9 medium; pfu, plaque-forming unit; λ^VIR^, virulent mutant of phage λ.

To test the validity of the model predictions, we performed serial transfer experiments with 10 independent cultures of λ-susceptible *E*. *coli* MG1655 mixed with an obligatory lytic mutant of phage λ (λ^VIR^) in LB and 12 in maltose (500 μg/mL)-limited M9 medium (M9M). The density of bacteria in LB was at the level observed for *E*. *coli* MG1655 grown in the absence of phage by the end of the first transfer (Fig 2A). The phage was stably maintained in all 10 experimental cultures at densities on average an order of magnitude lower than the density of bacteria (Fig 2B). Similarly, in parallel experiments with 12 independent serial transfer cultures of MG1655 and λ^VIR^ in M9M, the density of bacteria in all 12 cultures was comparable to that of the phage-free controls (Fig 2C) after the first couple of transfers. With respect to the maintenance of the phage, however, there were two qualitatively distinct outcomes. As was observed for all 10 LB cultures, in 7 of the 12 M9M cultures, the phage was maintained until the end of the experiment (Fig 2D). However, in the remaining five, the density of phage declined and the phage were lost (Fig 2D). This difference in outcomes can be explained by the fact that *E*. *coli* can become resistant to λ^VIR^ by mutations affecting the expression or structure of the maltose porin protein LamB, which, besides acting as the primary receptor for λ adsorption, serves as a transporter of maltose across the cytoplasmic membrane [40]. By performing these λ^VIR^ phage population dynamic/evolution experiments in maltose-limited medium, we are likely constraining the space of available resistance-conferring mutations (note that not all mutations conferring resistance to λ^VIR^ make *E*. *coli* unable to grow on maltose as the sole carbon source (Mal− phenotype) [40]).

To confirm that λ^VIR^-resistant *E*. *coli* evolved in the above cultures (Fig 2), we picked 10 colonies from each of the sampling plates used to estimate the density of bacteria at the seventh transfer. We streak purified these colonies and, by spotting phage lysates on soft agar lawns prepared from those colonies, we tested for their susceptibility to the ancestral λ^VIR^ as well as the coexisting phage. As measured by the ability to form plaques, all of the bacteria isolated from all 10 LB cultures, as well as all 12 M9M cultures, were resistant to both the ancestral phage and the coexisting phage. Moreover, the phage isolated from all cultures under both conditions were not able to form plaques on lawns of *lamB* and *malT* knockout mutants, both of which are resistant to wild-type λ^VIR^ (MalT is a positive regulator of LamB expression [41]). These results strongly indicate that while bacteria resistant to λ^VIR^ evolved in all experimental cultures, host range λ^VIR^ mutants capable of replicating on the resistant cells did not evolve in any. Coevolution is thus unlikely to explain the maintenance of the phage in these cultures, which, as we show, are dominated by bacteria upon which the phage cannot replicate.

As discussed above, resistance to λ^VIR^ is often, albeit not always, associated with loss of the ability to utilize maltose as a carbon source [40]. To check for the nature of the mutations that emerged in our experiments, we picked five colonies isolated from each replicate LB experiment at different serial transfers and tested the ability of these colonies to grown on minimal maltose plates. We observed that some of these λ^VIR^-resistant colonies were clearly Mal+ (confluent growth), some were clearly Mal− (no growth), and some produced a few Mal+ colonies (partial growth) (S1 Fig). Mal+ and Mal− colonies were found in most cultures at most time points (S1 Table), which suggests that the cultures were genetically heterogeneous.

To test whether reversions from λ^VIR^-resistance to susceptibility can be directly detected in these experiments, we picked five colonies isolated from each of the 10 LB cultures at the end of transfers one and nine. By streaking on LB agar for three passages, we "purified" these clones and thereby put them through single-cell bottlenecks. All 50 colonies tested from the first transfer as well as all 50 colonies tested from the last transfer were resistant to λ^VIR^. The maltose fermentation phenotype, on the other hand, was not equally stable. Fifteen resistant clones from the first transfer and nine from the last transfer produced clones that we characterized as partial growth, i.e., produced a few colonies in the maltose minimal agar. We isolated nine and six of these “partial growth” colonies from the first and last transfer, respectively, and tested for their resistance to wild-type λ^VIR^. All 15 colonies were susceptible to λ^VIR^.

#### Leaky resistance as a mechanism responsible for the maintenance of the phage in populations dominated by resistant bacteria

The “leaky resistance” hypothesis predicts the existence of λ^VIR^ -resistant bacterial mutants that revert from resistance to susceptibility at a high rate. As a consequence of this high reversion rate, λ^VIR^ should be able to become established and be maintained in populations initiated with bacteria already resistant at the beginning of the experiment (as opposed to experiments shown in Fig 2, in which we initiated the experiments with susceptible bacteria and let resistance evolve). To determine the validity of this prediction, we (i) explored whether λ^VIR^ can become established and be maintained in initially monoclonal populations of 12 independently isolated mutants resistant to λ^VIR^ and (ii) used a combination of modeling and experiments to estimate the actual rates of transition from resistance to susceptibility for these 12 independently isolated λ^VIR^- resistant mutants.

(i) λ^VIR^ can become established and maintained in initially monoclonal populations of resistant bacteria. We isolated 12 independent λ^VIR^-resistant mutants (designated W1–W12) of *E*. *coli* MG1655 and tested whether λ^VIR^ can become established and be maintained in serial passage cultures initiated with each of these resistant mutants. The 12 mutants were selected on LB soft agar plates from 12 independently grown cultures. Adsorption experiments revealed that none of the 12 mutants adsorbed phage at a detectable rate (S2 Fig). Because most of these mutants were unable to grow on M9M (with the exception of W2, W5, and W12), we only used LB in these and subsequent experiments.

**Fig 3:**
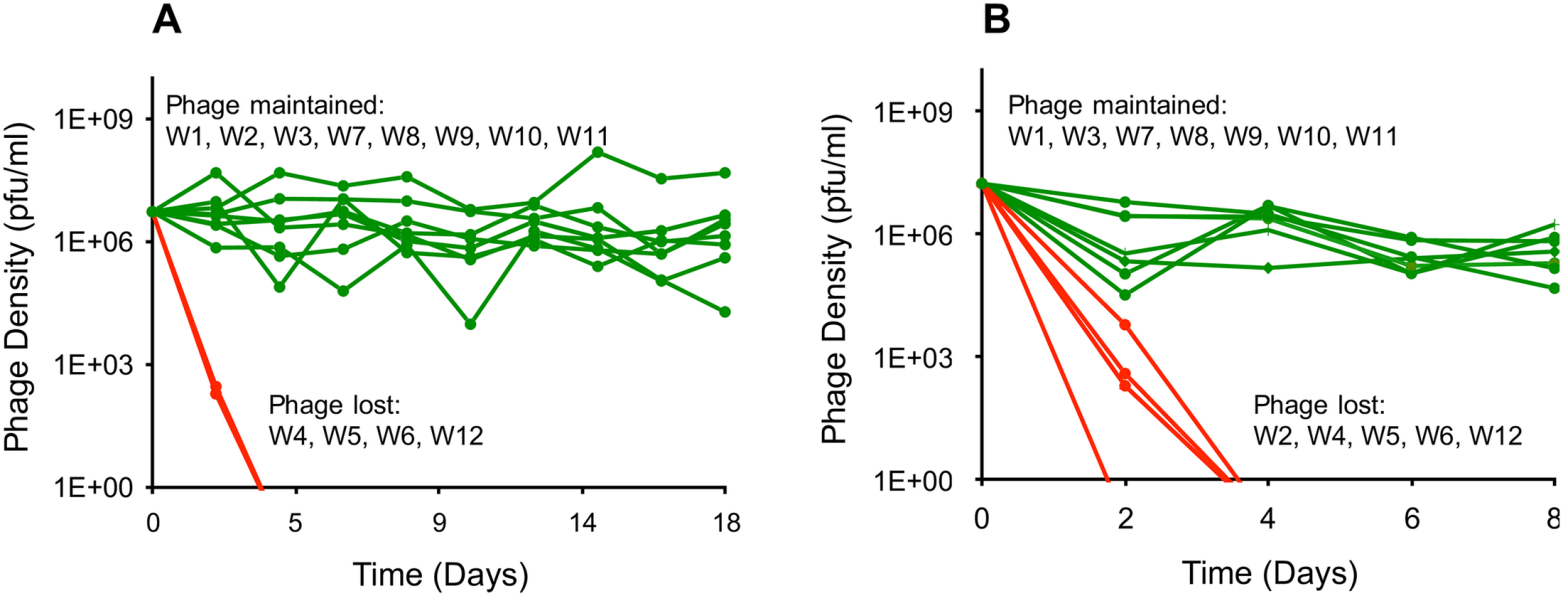
Changes in phage densities in initially monoclonal bacterial cultures initiated with resistant bacteria. **(A)** Changes in λ^VIR^ densities in 12 serial transfer cultures initiated with 12 independently isolated λ^VIR^-resistant mutants. Green lines correspond to cultures in which the phage was maintained; red lines correspond to cultures in which the phage was lost. **(B)** An independent replica of the experiment shown in (A) with 8 instead of 18 transfers. Underlying data can be found in S1 Data. pfu, plaque-forming unit; λ^VIR^, virulent mutant of phage λ.

While in 4 of the 12 cultures, the phage were lost by the fourth transfer, in the remaining 8, the phage were maintained in a seemingly stable state for 18 transfers (Fig 3A). An independent replicate of this experiment with the same 12 mutants lasting for 8 transfers yielded comparable results, with the phage being maintained in 7 out of 12 cultures (Fig 3B). Except for one mutant (W2), the results for different mutants were qualitatively consistent between the two replicates. These results indicate that the ability to support phage maintenance is genetically determined. In all cultures in which the phage were maintained, bacteria isolated at the end of the experiment were resistant to the ancestral λ^VIR^ phage as well as the coexisting phage. The latter suggests that the phage were maintained without the emergence of host range phage mutants capable of replicating on the resistant bacterial cells.

(ii) The rate of transition from resistance to susceptibility is in the range necessary for phage maintenance. The results of the simulations shown in Fig 1C suggest that, if the phage are to be maintained in populations dominated by resistant bacteria because of the transition from resistance to susceptibility, the rate of this transition (*μ*_*R*_) has to be of the order of 10^−5^ (per cell per hour) or higher. To experimentally estimate this rate, we devised a method that allowed for positive selection of λ^VIR^-susceptible bacteria from the dominant λ^VIR^-resistant population. We did this by employing a genetically marked temperate (wild-type) phage λ (λ^KAN^) [42], which can integrate into a bacterium’s genome and form lysogens that are resistant to kanamycin. Assuming that temperate λ are unable to infect and form lysogens on *E*. *coli* resistant to λ^VIR^ but can infect and form lysogens on bacteria that are λ^VIR^-susceptible, we can estimate the size of the λ^VIR^-susceptible subpopulation based on the rate of formation of λ^KAN^ lysogens.

**Fig 4:**
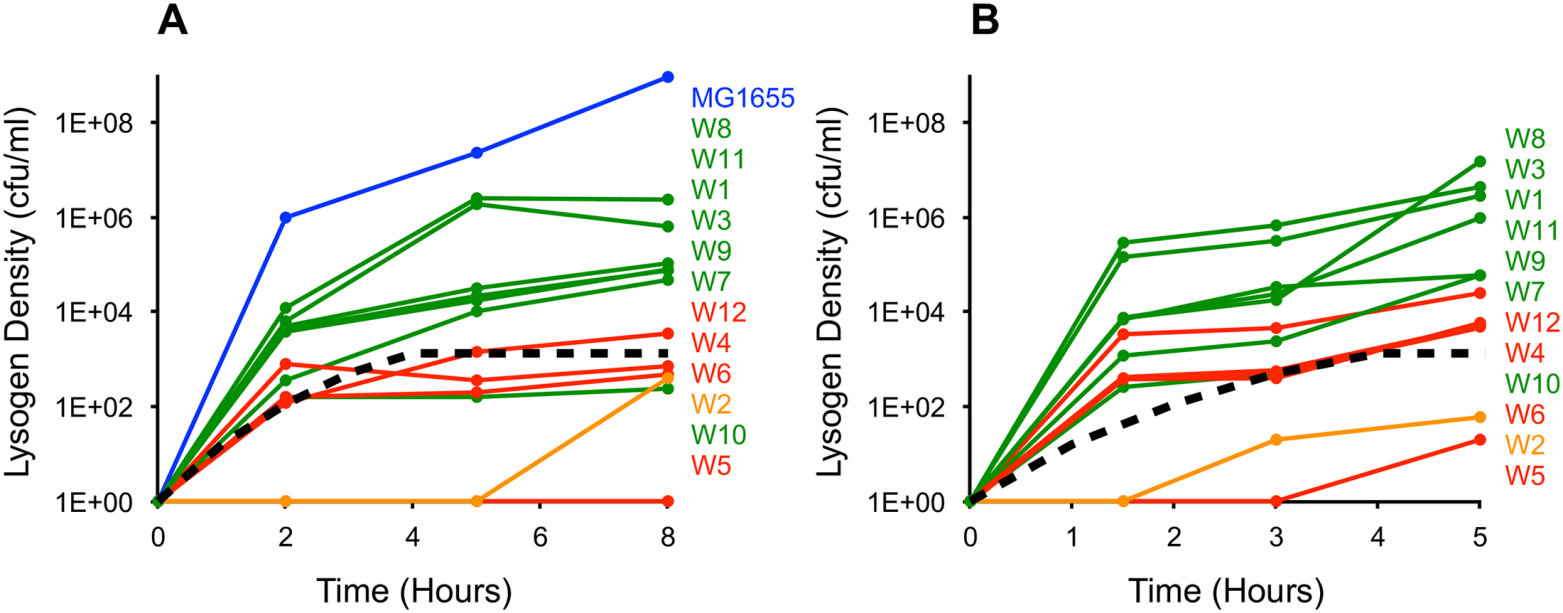
Dynamics of λ^KAN^ lysogen formation with 12 independently isolated λ^VIR^- resistant *Escherichia coli* mutants. **(A)** and **(B)** are independent replicates. Green lines correspond to mutants that maintained λ^VIR^ in Fig 3A and 3B. Red lines correspond to mutants that did not maintain the phage. W2 is in orange, as it yielded different outcomes. The black dashed lines correspond to numerical solutions of simulations, assuming the rate of transition in the range predicted to be necessary to maintain λ^VIR^ (*μ*_*N*_ = 10^−5^). The remaining parameters were identical to those used in Fig 1, with a probability of lysogeny of λ = 0.02. The labels at the right are ordered according to the final density of lysogens produced. The blue line shows dynamics of lysogen formation in a fully susceptible strain. The experiment with a fully susceptible strain was only performed in one of the two replicates. The final number of λ^KAN^ lysogens produced significantly correlated with the ability of λ^VIR^ to be maintained on these mutants (*p* = 7.9 × 10^−4^ with 24 observations and 22 degrees of freedom). The *p*-value was calculated using logistic regression, with the log-transformed number of λ^KAN^ lysogens at the end of the experiment as a continuous independent variable and the ability to maintain λ^VIR^ (yes/no) as a binary dependent variable. In these statistical tests, it was assumed that W2 cannot maintain λ^VIR^. Assuming that it can maintain the phage still yielded a significant correlation (*p* = 0.005). Underlying data can be found in S1 Data. cfu, colony-forming unit; λ^KAN^, genetically marked temperate (wild-type) phage λ; λ^VIR^, virulent mutant of phage λ.

For this, we inoculated cultures of the 12 λ^VIR^-resistant (W1–W12) mutants with λ^KAN^ and followed the dynamics of lysogen formation by estimating the number of kanamycin-resistant lysogens at regular time intervals. The results of two independent replicate experiments are presented in Fig 4A and 4B. Except for W5, all λ^VIR^-resistant strains produced λ^KAN^ lysogens at densities higher than the detection limit (about 10 cells per mL), although they did so at different rates and yielded different final lysogen densities. In general, mutants whose populations were able to maintain λ^VIR^ in serial transfers (green curves) produced significantly more λ^KAN^ lysogens than mutants whose populations did not allow for λ^VIR^ maintenance (red curves). This result was observed in both independent replicate experiments. An exception to this trend was mutant W10, which did maintain λ^VIR^ but produced λ^KAN^ lysogens at a low rate in both replicate experiments.

We compared the experimental results with the expected dynamics of lysogen formation that assumed the rate of transition from resistance to susceptibility predicted as necessary for phage maintenance (*μ*_*R*_ = 10^−5^ per cell per hour). These anticipated dynamics were obtained as numerical solutions to a model of the population dynamics of temperate phage and resistant bacteria [43] (Eqs 5–9).

$$\frac{dR}{dt}\;=\;\underset{resource\;uptake}{\underbrace{-e\cdot \psi (R)\cdot (v\cdot N+v_{r}\cdot N_{r}+v_{l}\cdot L)}}$$ (5)

$$\frac{dN}{dt}\;=\;\underset{growth}{\underbrace{v\cdot \psi (R)\cdot N}}-\underset{phage\;lysis}{\underbrace{\delta \cdot \psi (R)\cdot N\cdot P}}+\underset{transition}{\underbrace{\left(\mu_{R}\cdot N_{R}-\mu_{N}\cdot N)\cdot \psi (R\right)}}$$ (6)

$$\frac{dN_{R}}{dt}\;=\;\underset{growth}{\underbrace{v_{R}\cdot \psi (R)\cdot N_{R}}}+\underset{transition}{\underbrace{\left(\mu_{N}\cdot N-\mu_{R}\cdot N_{R})\cdot \psi (R\right)}}$$ (7)

$$\frac{dL}{dt}\;=\;\underset{growth}{\underbrace{v_{L}\cdot \psi (R)\cdot L}}+\underset{lysogenisation}{\underbrace{\delta \cdot \lambda \cdot \psi (R)\cdot N\cdot P}}-\underset{induction}{\underbrace{\iota \cdot \psi (R)\cdot L\cdot \beta }}$$ (8)

$$\frac{dP}{dt}\;=\;\underset{phage\;lysis}{\underbrace{\delta \cdot \psi (R)\cdot N\cdot P\cdot (\beta -1)\cdot (\lambda -1)}}+\underset{induction}{\underbrace{\iota \cdot \psi (R)\cdot L\cdot \beta }}-\underset{adsorption\;to\;lysogens}{\underbrace{\delta \cdot \psi (R)\cdot L\cdot P}}$$ (9)

In this mathematical model, *ψ*(*R*) = *R*/(*k* + *R*), *P* is the density of the temperate phage (particles per mL), *L* is the density of lysogens (cells per mL), *λ* is the probability that, upon adsorption of a temperate phage, a lysogen will be formed, (1−*λ*) is the probability that the infection will be lytic and *ι* is the rate of spontaneous induction. The remaining variables and parameters are identical to those defined for the lytic phage model presented above (Eqs 1–4).

In both replicates, the majority of mutants on whose populations λ^VIR^ was maintained produced lysogens at a rate higher than the model approximation (Fig 4A and 4B), and the final number of λ^KAN^ lysogens produced significantly correlated with the ability of these mutants to maintain λ^VIR^. This result is consistent with what would be anticipated if mutants maintaining λ^VIR^ reverted to susceptibility at a sufficiently high rate.

From each replicate experiment shown in Fig 4, we picked five λ^KAN^ lysogens and tested their susceptibility to λ^VIR^. If the cells that formed these lysogens transitioned from resistance to susceptibility by genetic mechanisms, we would expect these colonies to be susceptible to λ^VIR^ (note that unlike wild type λ, the virulent mutant λ^VIR^ is immune to superinfection exclusion and can infect λ lysogens). The results of this experiment are presented in Table 1. While λ^KAN^ lysogens formed by some mutants were susceptible to λ^VIR^, others remained resistant. Thus, while some mutants transition from resistance to susceptibility by genetic reversion, other mutants seem to produce cells that are phenotypically susceptible but genetically resistant. Interestingly, we observed no clear correlation between the ability of these mutants to maintain λ^VIR^ and the number of susceptible lysogens produced, which suggests that either genetic or phenotypic transition can lead to phage maintenance, given that it occurs at a sufficiently high rate.

**Table 1:** Numbers of lysogens susceptible to λ^VIR^. Five λ^KAN^ lysogenic colonies formed by the twelve resistant mutants were tested for their susceptibility to λ^VIR^, as evaluated by a spot test assay. The rows shaded in green correspond to strains that maintain λ^VIR^, whereas those shaded in red correspond to strains in which the phage was lost. For W2, different results were obtained in two replicate experiments. For W5, no lysogens were produced and their susceptibility thus could not be assayed.

Strain #	Replica A	Replica B
W1	3/5	5/5
W2	5/5	3/5
W3	4/5	5/5
W4	0/5	0/5
W5		
W6	0/5	0/5
W7	0/5	0/5
W8	5/5	5/5
W9	5/5	3/5
W10	0/5	0/5
W11	0/5	0/5
W12	5/5	5/5

Abbreviations: λ^KAN^, genetically marked temperate (wild-type) phage λ; λ^VIR^, virulent mutant of phage λ.

#### Genetic determinants of leaky resistance

**Fig 5:**
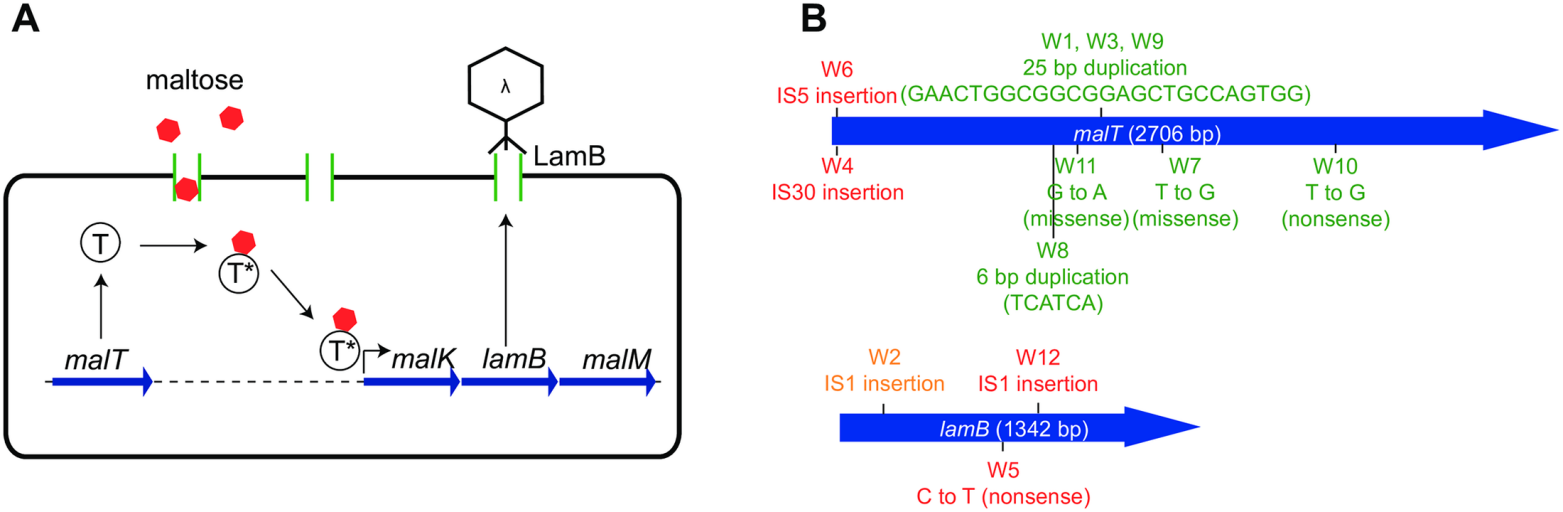
The genetic basis of leaky resistance. **(A)** Schematics of *lamB* regulation. Upon binding maltose, MalT serves as a transcriptional activator of the operon containing *lamB*, which encodes the phage λ receptor protein LamB. **(B)** Mutations identified in the 12 λ^VIR^-resistant strains and their location. Mutations listed in green were found in strains that allowed for stable maintenance of λ^VIR^, whereas those listed in red were found in strains in which λ^VIR^ was not maintained. W2, which allowed for phage maintenance in one of two replicate experiments, is shown in orange. λ^VIR^, virulent mutant of phage λ.

Why do the 12 independently isolated λ^VIR^ resistant strains (W1–W12) differ in terms of their capacity to maintain λ^VIR^ as well as their capacity to form λ^KAN^ lysogens? To answer this question, we identified the mutations responsible for their resistance phenotypes by sequencing the *lamB* and *malT* genes, the two loci known to cause resistance to phage λ [41]. The *lamB* gene codes for a porin protein involved in transport of maltose and other maltodextrins, which also acts as the receptor for λ attachment. Its expression is positively regulated by the transcriptional activator MalT, in whose absence *lamB* is not expressed [44] (Fig 5A). As a consequence, both *malT* and *lamB* mutants are resistant to infection by phage λ.

All 12 independently isolated λ^VIR^-resistant strains (W1–W12) carried a mutation in either *lamB* or *malT*, although the exact locations and the nature of these mutations differed among the isolated strains (Table 1). We identified a spectrum of mutations, including single point mutations (W5, W7, W10, W11), short amplifications (W1, W3, W8, W9), and disruption of coding sequences by Insertion sequence (IS) elements (W2, W4, W6, W12). Except for a 25-bp-long amplification inside the *malT* coding sequence, which was identified in three independently picked mutants (W1, W3, W9), all mutations causing the λ^VIR^-resistant phenotype were unique in the set (Fig 5B).

As shown in Fig 5B, none of the λ^VIR^-resistant strains carrying mutations in the *lamB* gene could maintain λ^VIR^ (marked in red). In contrast, with the exception of two mutants (W4 and W6) harboring IS elements, the majority of λ^VIR^-resistant strains carrying mutations in the *malT* gene did allow for stable λ^VIR^ maintenance. These results show that both the location and nature of a mutation causing resistance are important determinants of phage maintenance, as they interfere in different ways with LamB function.

In Table 2, we summarize the data presented in Figs 3, 4 and 5 to help us better understand the connection between the nature of the mutations causing resistance and their effects on phage maintenance. The rate at which these 12 λ^VIR^-resistant mutants formed lysogens upon infection by λ^KAN^ was the best predictor of whether or not λ^VIR^ can be maintained on these strains. As can be seen in Table 1, lysogens formed by mutants with short duplications and insertion elements were λ^VIR^ susceptible, indicating that these lysogens formed on cells that genetically reverted from resistance to susceptibility. For these mutations, genetic reversion is not unexpected, given the relatively high rates of loss of duplications and excision of insertion elements in bacterial genomes [45,46]. We confirmed that the genetic reversions leading to formation of λ^VIR^-susceptible lysogens were indeed genetic by sequencing the *lamB* and *malT* genes of five λ^KAN^ lysogens formed on strains W1 (four of which were λ^VIR^ susceptible), W2 (all were λ^VIR^ susceptible), and W7 (all were λ^VIR^ resistant). The sequences have revealed that the genetic reversion to susceptibility was in all cases caused by loss of the original mutation. In contrast, the lysogens that remained λ^VIR^ resistant still carried the original mutation and no additional mutations were identified.

**Table 2:** Phenotypic and genotypic characterization of the 12 resistant mutants used in this study. Rows shaded in green correspond to strains that maintain λ^VIR^, whereas those shaded in red correspond to strains in which the phage was lost. For W2, shown in orange, different outcomes were obtained in two replicate experiments.

Strain #	Lysogen Production Rate	Lysogen Susceptible to λ^VIR^	*lamB* Sequence	*malT* Sequence
W1	high	8/10	wt	GAACTGGCGGCGGAGCTGCCAGTGG duplication at 1,022
W2	low	8/10	IS1 at 167	wt
W3	high	9/10	wt	GAACTGGCGGCGGAGCTGCCAGTGG duplication at 1,022
W4	low	0/10	wt	IS30 at 19
W5	no		C607T nonsense	wt
W6	low	0/10	wt	IS5 at 18
W7	high	0/10	wt	T1232G missense
W8	high	10/10	wt	TCATCA duplication at 856
W9	high	8/10	wt	GAACTGGCGGCGGAGCTGCCAGTGG duplication at 1,022
W10	low	0	wt	T1874G nonsense
W11	high	0	wt	G914A missense
W12	low	10/10	IS1 at 752	wt

Abbreviations: IS, Insertion sequence; wt, wild-type; λ^VIR^, virulent mutant of phage λ.

The three strains that carried missense point mutations in the *malT* gene (W7, W10, W11) all formed lysogens, although, surprisingly, the lysogenic colonies remained λ^VIR^-resistant. This result indicates that among these resistant mutants, there are individual cells that are phenotypically susceptible, although colonies formed by these strains behave as if they were genetically resistant. Such phenotypic susceptibility could result from spontaneous *lamB* expression in a subpopulation of bacteria because of low-level *malT* expression. Switching between phenotypes because of the stochastic nature of gene regulation was previously proposed to occur for a number of molecular systems [47], including the maltose operon [48,49].

To test the generality of our results, we repeated the experiments with the strain of *E*. *coli* B employed in [50] rather than K12, as used in the preceding experiments, and performed these experiments in glucose-limited M9 medium rather than LB and M9M used in the preceding. In these experiments, λ^VIR^ was readily maintained in cultures initiated with spontaneous λ^VIR^-resistant mutants bearing the 25-base duplication in *malT*, also found in the mutants W1, W3, and W9 (Fig 5). In contrast, a λ^VIR^-resistant mutant carrying a nonsense *malT* mutation was unable to maintain the phage (S3 Fig).

#### Direct estimation of the rate of genetic transition from resistance to susceptibility

**Fig 6:**
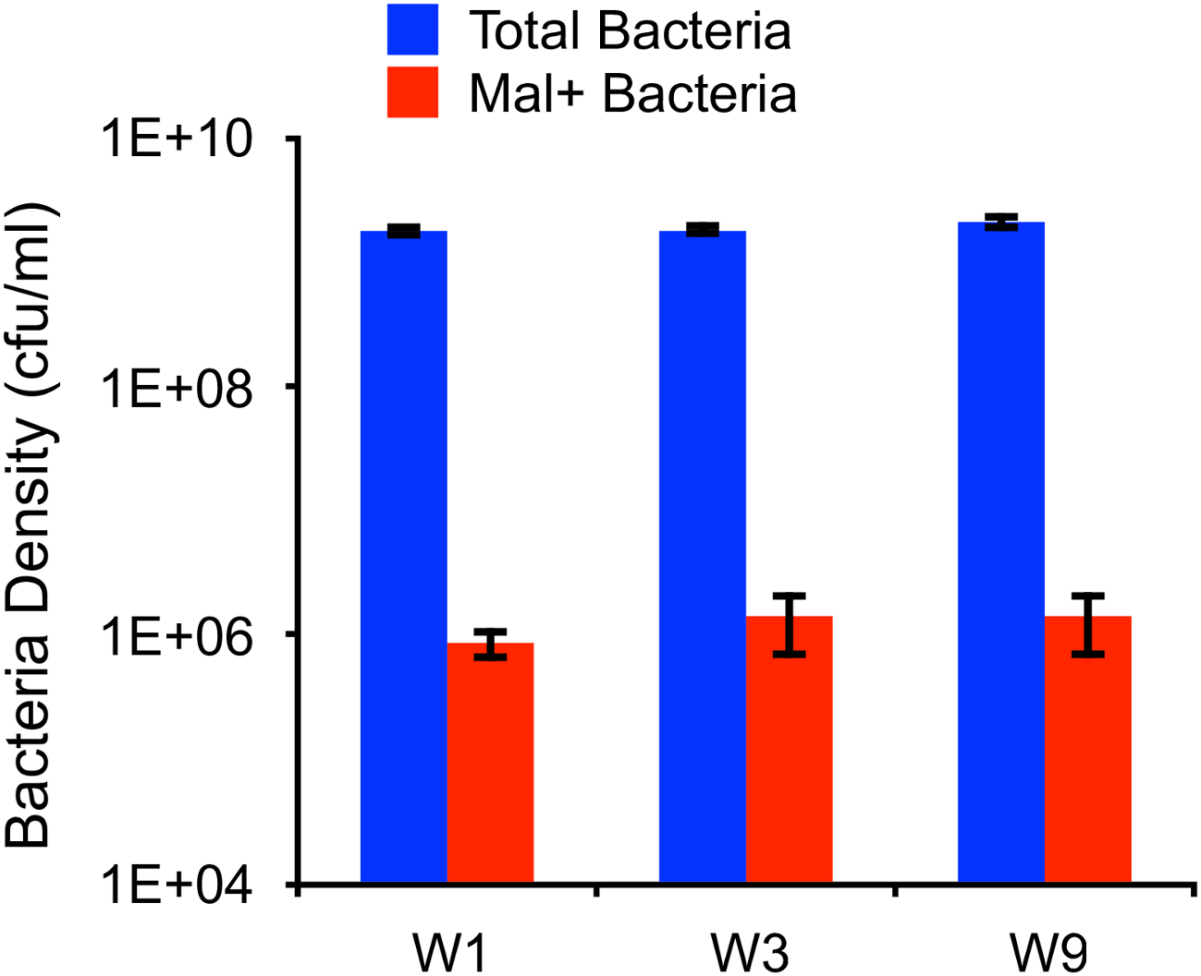
Densities and rate of reversion from Mal− to Mal+ in strains W1, W3, and W9. Mean and standard error in total bacterial density (*n* = 3) and in the number of Mal+ mutants (*n* = 12) are shown. Underlying data can be found in S1 Data. cfu, colony-forming unit; Mal+, phenotype characterized by the ability of bacteria to growth on maltose as a sole carbon source.

The most common mutation found in our experiments was a short (25-bp) duplication in the *malT* gene, which was present in 3 out of the 12 independently isolated λ^VIR^-resistant mutants (W1, W3, W9). As shown in Figs 3 and 5, respectively, these resistant mutants can support the maintenance of λ^VIR^ and produce λ^VIR^-susceptible lysogens that have lost the original mutation. Furthermore, while the original W1, W3, and W9 strains are Mal−, the λ^KAN^ lysogens formed by these strains are Mal+. These observations point towards a high rate of genetic reversion from resistance to susceptibility. Using the change in the Mal phenotype, we performed fluctuation tests and estimated the rate of this genetic transition directly by estimating the density of Mal+ revertants on M9M agar plates. The total number of cells plated and the number of Mal+ revertants obtained are presented in Fig 6. We used these data and the method described in [51] to estimate the corresponding mutation rates. The estimated mutation rates for reversion to Mal+ ranged from 2.3×10^−5^ to 2.7×10^−5^ per generation. If we assume a generation time of 30 minutes, the resulting rate of reversion (about 5×10^−5^ per cell per hour) is above the estimated transition rate necessary for phage maintenance (*μ*_*N*_ = 10^−5^ per cell per hour) required by the “leaky resistance” hypothesis.

### Alternative mechanisms responsible for the maintenance of the phage in populations dominated by resistant bacteria

In addition to the “leaky resistance” mechanism described here, at least three other mechanisms could, in principle, lead to stable phage maintenance in populations dominated by bacteria upon which the phage cannot replicate. While these mechanisms are not mutually exclusive and could, in principle, act together in some populations, in the following section we describe reasons why these alternative mechanisms are unlikely to play a significant role in our experiments.

#### Cost of resistance

In cases in which resistance to phage is associated with a significant fitness cost, phage can maintain their population in an apparently stable state in a population dominated by bacteria resistant to the phage because of the existence of a subpopulation of susceptible cells that persist as a result of their fitness advantage [18,22,52–55]. While we cannot fully rule out that fitness cost contributes to the dynamics observed in Fig 2, it is not sufficient to explain the phage maintenance observed in these experiments for two reasons. First, our model predicts that the fitness cost of resistance required for the stable maintenance of the phage in populations dominated by resistant cells would have to be in excess of 20% (S4 Fig) (also see [31]). We observed no evidence for a fitness cost even close to this magnitude, at least not as measured by maximum growth rates of the 12 independently isolated resistant mutants, as compared to the ancestral susceptible strain (S5 Fig). Second, we observe continuous phage maintenance in populations initiated solely with resistant bacteria (Fig 3), in which, in the absence of transition from resistance to susceptibility, no susceptible bacteria should be present.

#### Coevolutionary arms race

After bacteria evolve resistance to phage, the phage can respond by evolving host range mutations that overcome this resistance [35]. If coevolution were the main mechanism enabling phage maintenance in our experiments, the phage isolated in later transfers of the cultures in which they were maintained should form plaques on lawns of resistant bacteria from earlier transfers. This was not the case in any of our experiments. Moreover, the phage isolated from these serial transfer cultures were unable to form plaques on lawns of *E*. *coli* MG1655 with deletions in the *lamB* or *malT* genes. Deletions in either of the two genes render the bacteria resistant to infection by wild-type λ [49].

#### Habitat heterogeneity

Phage can also be maintained by replicating on a minority population of susceptible bacteria shed from the walls of the culture vessels [36]. While we cannot fully exclude some contribution of this mechanism to the stable maintenance of the phage observed in our experiments, we expect this contribution is at best small. In contrast to the original study by Schrag and Mittler [36], which provided theoretical and experimental evidence in support of this hypothesis as an explanation for the maintenance of phage in continuous culture populations dominated by resistant bacteria, in our experimental serial transfer regimen, only the liquid portion of the population is transferred. Such serial transfer provides strong selection against sessile bacterial wall growth. Even if susceptible cells established a population on the walls of the culture vessels, in the course of the serial transfers that population would be diluted out.

## Discussion

The results of these experiments with *E*. *coli* and λ^VIR^ are consistent with other laboratory experiments with lytic phage and bacteria in liquid culture [21–27,31,34]. When *E*. *coli* MG1655 and λ^VIR^ are mixed in serial transfer culture, within short order, λ^VIR^-resistant *E*. *coli* emerge and ascend to densities similar to that of phage-free, resource-limited cultures. Despite the evolution of bacteria upon which they cannot replicate, in the majority of these experiments (7 out of 12 in M9M and 10 out of 10 in LB medium), λ^VIR^ continues to be maintained in these cultures in a seemingly stable state. The stable maintenance of the phage following the evolution of resistance is not anticipated from a simple, mass action model of the population dynamics of phage and bacteria with the bacterial growth and phage replication parameters in the range estimated.

While our analysis and experiments cannot fully rule out the contribution of other mechanisms, such as fitness cost of resistance phenotypes, they provide compelling support for the hypothesis that the dominant mechanism responsible for the maintenance of λ^VIR^ in these experiments can be attributed to a high (10^−5^ per cell per hour or greater) rate of transition from λ^VIR^ resistance to λ^VIR^ susceptibility, a phenomenon we term “leaky resistance.” We use the word “transition” rather than “mutation” for the change in state because our results indicate that, in addition to genetic changes, this transition can also be due to phenotypic effects. It should be noted that the “leaky resistance” mechanism considered here is different than what Bull and colleagues called phenotypic resistance [56]. In their study, the dominant population of bacteria, while having a reduced adsorption rate, appeared sensitive to the phage by standard plaque-forming tests. In contrast, the colonies formed by bacteria dominating the cultures in our experiments tested as resistant and no apparent adsorption could be observed. Although it is possible that the reverted λ^VIR^-susceptible bacteria have lower adsorption rates as compared to the wild type, our experiments indicate that these bacteria would still have to be present as a subpopulation, rather than dominate the cultures.

### Implications and caveats

If the population and evolutionary dynamics of bacteria and phage in natural communities reflect what is observed in experimental populations (like those studied here and many other investigations), we would have to conclude that viruses play little or no role in regulating the population densities of their host bacteria. On the other hand, bacterial and phage populations in nature are complex systems. As a result of environmental and population heterogeneity, different mechanisms allowing for phage maintenance in simple experimental cultures likely interact to produce the ecological and evolutionary dynamics observable in natural populations. Indeed, evidence of adaptation of viruses to resistant hosts [28,57] as well as coevolutionary dynamics [58,59] can frequently be observed in natural populations. The mechanism of “leaky resistance” described here could allow phage to persist in a population dominated by resistant bacteria for periods of time necessary for host range mutations to appear [49], thus playing an important role in facilitating coevolutionary dynamics.

Loss of immunity was recently proposed as the mechanism responsible for stable coexistence of a lytic phage 2972 and *Streptococcus thermophilus* with CRISPR-Cas–mediated immunity to this phage [31]. The concept of immunity differs from that of resistance in that immune bacteria neutralize the phage genetic material once it enters the cell, whereas resistant bacteria prevent injection of the phage genetic material in the first place. The model Weissman and colleagues used in this study was similar to that employed here and also derived from that in [19]. If we substitute resistance for immunity, the conclusions of Weissman and colleagues are analogous to those presented here; if the rate of transition from phage resistance to susceptibility is sufficiently high, lytic phage will be stably maintained in communities dominated by bacteria upon which the phage cannot replicate. The rate of transition from immunity to susceptibility was not directly measured in Weissman and colleagues [31] but rather inferred from the rate of loss of CRISPR-Cas in plasmid transfer experiments, with *Staphylococcus epidermidis* bearing CRISPR-Cas–mediated immunity to the plasmid [60]. In this study, we provide direct evidence showing that a high rate of transition from phage resistance to susceptibility is the main mechanism accounting for stable maintenance of phage in populations dominated by bacteria upon which they cannot replicate.

Jacque Monod is reported to have quipped: “What is true for *E*. *coli* is true for elephants.” While we certainly appreciate this bacteriocentric form of inductive inferences, we will not argue that the results reported with our simple models and experiments with *E*. *coli* and λ^VIR^ are general for all bacteria and phage in laboratory culture, much less in natural communities. On the other hand, high rates of transition between resistant and susceptible states were previously observed for *Pseudomonas aeruginosa* grown in the presence of phage PP7 [61]. For *P*. *syringae*, it was shown that some resistant mutants can maintain populations of phage ϕ6 stably despite the absence of a significant fitness cost and with no evidence of coevolution [62]. Is a high rate of transition from resistance to susceptibility observed here for *E*. *coli* and λ^VIR^ responsible for the stable maintenance of viruses in these experiments and those with other bacterial and phage species? This leaky resistance hypothesis can be readily tested with any species of bacteria and phage that can be cultured in vitro. The first step in testing this hypothesis is to establish experimental cultures in which phage are maintained despite resistant cells emerging and dominating the bacterial population. If leaky resistance is the mechanism responsible for the maintenance of the phage in these experiments, without subsequent host range mutation, the phage should be able to become established and be maintained in populations derived from these resistant clones.

Particularly striking in this study is the observation that genetic changes are occurring at rates several orders of magnitude greater than that anticipated by classical point (base change) mutation [63]. High rates of mutation caused by insertion and removal of either short duplications or IS elements can thus play an important role in the ecology and evolution of bacteria. Recent experiments also support this perspective [34,64].

## Materials and methods

### Growth media and strains

Bacterial cultures were grown at 37 °C in either M9M (M9 salts [248510, Difco] supplemented with 0.4% maltose [6363-53-7, Fisher Scientific], 1 mM MgSO_4_, 0.1 mM CaCl, and 0.2% Thiamine [B1]) or LB (244620, Difco). All *E*. *coli* strains used in our experiments were wild-type K12 derivatives of the parent strain MG1655. The K12 and *E*. *coli* B6 strains used to test for evolution of host range mutations (K12 *malT*^*−*^, K12 *ompF*^*−*^, K12 *lamB*^*−*^, B6 *lamB*^*−*^) were obtained from [49]. Phage lysates were prepared from single plaques at 37 °C in LB medium alongside wild-type MG1655. Chloroform was added to the lysates and the lysates were centrifuged to remove any remaining bacterial cells. The λ^VIR^ strain used in these experiments was obtained from Sylvain Moineau. The construction of λ^KAN^ is described in [42]. The 12 resistant mutants (W1–W12) were picked from 12 LB soft agar plates, in which independently grown cultures of *E*. *coli* MG1655 were plated as a lawn and λ^VIR^ was spotted on top of the plates.

### Sampling bacterial and phage densities

Bacteria and phage densities were estimated by serial dilution in 0.85% saline followed by plating. The total density of bacteria was estimated on LB hard (1.6%) agar plates. To estimate the densities of λ^KAN^ lysogens, cultures were plated on LB agar with 25 μg/mL kanamycin (AppliChem Lot# 1P0000874). To estimate the densities of free phage, chloroform was added to suspensions before serial dilution. These suspensions were mixed with 0.1 mL of overnight LB-grown cultures of wild-type MG1655 (about 5×10^8^ cells per mL) in 3 mL of LB soft (0.65%) agar and poured onto semihard (1%) LB agar plates. Bacteria were tested for resistance by spotting about 25 μL of a lysate (>10^8^ plaque-forming units [pfu]/mL) on LB soft agar lawns with about 10^8^ bacteria. Susceptibility to λ^VIR^ was noted as clear zones. Failure to see zones was interpreted as evidence for resistance. There is, however, a caveat to this procedure. If 1% or less of the cells in the lawn were susceptible to the phage, there would be no visible plaques (S6 Fig).

### Serial transfer experiments

All serial transfer experiments were carried out in 10-mL cultures grown at 37 °C with vigorous shaking. The cultures were initiated by 1:100 dilution from 2-mL overnight cultures grown from single colonies. Phage were added to these cultures to reach the initial density of approximately 10^6^ pfu/mL. At the end of each transfer, 100 μL of each culture were transferred into flasks with fresh medium (1:100 dilution). Simultaneously, 100-μL samples were taken for estimating the densities of colony-forming units (cfu) and pfu, as described above.

### Measuring the rate of λ^KAN^ lysogen formation

Overnight LB cultures of the W1–W12 strains were grown from single colonies. These cultures were diluted 1:100 in 2 mL of fresh LB and λ^KAN^ was added to reach the density of approximately 5×10^5^ pfu/mL. The dynamics of lysogen formation were followed by sampling 100 μL at regular intervals and plating the diluted samples at regular time intervals on LB agar with 25 μg/mL kanamycin.

### Estimating the rate of mutation from λ^VIR^ susceptibility to λ^VIR^ resistance and from Mal− to Mal+ (fluctuation experiments)

We estimated the rate of mutation to λ^VIR^ resistance as follows: 12 independent 10-mL cultures of MG1655, each initiated with about 1E4 cells, were grown overnight in LB to stationary phase. The next day, 100 μL of these overnight cultures was added to 3 mL LB and allowed to grow for 1 hour at 37 °C. One hundred milliliters of these cultures was added to 1 mL of a high titer (>10^9^ pfu/mL) λ^VIR^ lysate. The mixture was incubated for 45 minutes at 37 °C. While these cultures were incubating, the viable cell densities in their cultures were estimated by serial dilution and plating. After 45 minutes of incubation with the phage, 200 μL of the mixture was spread on LB agar to estimate the number of resistant bacteria. The estimate of the mutation rate was made according to the protocol described in [51], using the median number of mutants in the fluctuation experiment and the fraction of the total cultures plated.

We estimated the rate of mutation from Mal- to Mal+ as follows: for strains W1, W3, and W9 (all Mal−), 12 independent 2-mL LB cultures of each were initiated with about 1E4 cells in 12-well macrotiter plates. These cultures were grown overnight at 37 °C. Subsequently, 1 mL of these cultures was sampled, and the residual LB medium was removed by centrifugation. The pelleted cells were then resuspended in 1 mL of 0.85% saline. This process was repeated three times. The total cell densities of *E*. *coli* in these washed cultures were estimated by serial dilution and plating on LB agar. The washed cultures were then diluted 1/10 in saline, and 100 μL from each independent culture was spread onto M9M agar. The mutation rate to Mal+ was estimated with the protocol in [51], using the median cfu on the M9M agar and the fraction of the total culture plated.

### Identifying the mutations responsible for λ resistance

The *lamB* and *malT* genes were amplified from individual colonies using the fw_lamB, rv_lamB and fw_malT, rv_malT primers (Table 3), respectively, in a standard PCR reaction with Taq DNA Polymerase (Sigma). The PCR products were then purified using the GenElute Gel Extraction kit (Sigma) and sequenced. The *malT* gene was sequenced using primers fw_malT, rv_malT, s1_malT, s2_malT, s3_malT, s4_malT. The *lamB* gene was sequenced using primers fw_lamB, rv_lamB, s1_lamB, s2_lamB. This primer arrangement covers the sequence of both genes at least twice. Mutations were identified by comparing the sequences of the mutants to the corresponding sequences of wild-type *E*. *coli* MG1655.

**Table 3:** Primers used in this study.

Primer	Sequence
fw_malT	AATCTGATGAACATAAGGGAAAC
rv_malT	CGGTGCGGTTTAGTTTGA
s1_malT	TCTTCGCCGGTCACAC
s2_malT	CGTCTGGAAAACCTGCT
s3_malT	ATCGCCGGTCATTTGC
s4_malT	TTGAAAAGCGCCATTTTG
fw_lamB	ATCCCTTCCATTCGTCAAAA
rv_lamB	TACATTTGACAGCCGTTGTA
s1_lamB	CGCCATCAACCAGACGA
s2_lamB	TTAGAACTGGGTGTCGACT

### Parameter estimations

The parameters critical for the interaction of λ and *E*. *coli* used in this study were estimated in independent experiments in LB medium. The maximum growth rates of *E*.*coli* and the 12 resistant mutants were measured by Bioscreen as described in [10,65]. Phage burst sizes (β) were estimated with one-step growth experiments [66] in a manner similar to [67]. Adsorption of λ to *E*. *coli* was estimated as described in [19]. The procedure for estimating the probability of lysogeny and the rate of spontaneous lysogen induction are presented in [42].

## Supporting information

S1 Fig Results of a streak test for maltose fermentation by λ^VIR^-resistant *Escherichia coli*. Both Mal+ and Mal− phenotypes are observed. Some isolates show partial growth and produces a few small colonies. Mal, maltose phenotype; λ^VIR^, virulent mutant of phage λ.
(TIF)

S2 Fig Adsorption of λ^VIR^ to the 12 resistant *Escherichia coli* mutants. Densities of infective centers in two independent replicate experiments are shown. In each experiment, approximately 10^5^ pfu/mL were mixed with 10^8^ cfu/mL in 1 mL LB medium and the phage density was measured. After 25 minutes, the samples were chloroformed to kill all bacteria, including those that adsorbed phage, and the density of free phage was estimated by plating. In all samples, the bacterial density remained unchanged in the course of the experiment. Underlying data can be found in S1 Data. cfu, colony-forming unit; LB, Lysogeny broth; pfu, plaque-forming unit; λ^VIR^, virulent mutant of phage λ.
(TIF)

S3 Fig Changes in phage densities in monoclonal bacterial cultures initiated with different resistant mutants of *Escherichia coli* B. Serial transfer cultures were initiated with λ^VIR^-resistant *E*. *coli* B (REL 606) mutants carrying either a 25-bp duplication in the *malT* gene, or independently isolated SNP mutations in *malT* (frameshift, due to a T inserted between nucleotides 610 and 611) and *lamB* (nonsense C->T at nucleotide 883 that creates a stop codon in AA #295) genes. The black line shows phage densities estimated in a serial transfer experiment with no host bacteria. Data points represent means of four biological replicates. Error bars represent the standard error of the mean (*n* = 4). The experiments were performed in M9 minimal medium supplemented with 1 mg/mL glucose as the limiting carbon source. Underlying data can be found in S1 Data. λ^VIR^, virulent mutant of phage λ.
(TIF)

S4 Fig Simulation results: The effect of fitness cost of resistance on phage maintenance. The parameter W, fitness, is the ratio of the maximum growth rate of the resistant clone, relative to the susceptible (*v*_*R*_/*v*_*S*)_. In these simulations: *δ* = 2 × 10^−7^, *β* = 50, C = 1,000, *v* = 1, *d* = 0.01, *e* = 5 × 10^−7^, *k* = 1, *μ*_*N*_ = 5 × 10^−6^, *μ*_*R*_ = 0, ref = 10^2^. In **(A)** and **(B)**, the fitness cost of resistance is not sufficient to allow for phage maintenance. In **(C)** and **(D)**, the phage is maintained as a result of the high fitness cost. Underlying data can be found in S1 Data. *v*_*R*_, maximum growth rate of resistant strain; *v*_*S*_, maximum growth rate of susceptible strain.
(TIF)

S5 Fig Maximum growth rates of 12 independently isolated λ^VIR^-resistant mutants. Mean and standard errors of five replicas are shown. The λ^VIR^-susceptible ancestor strain is shown in black. **(A)** Maximum growth rates in M9 glucose (500 μg/mL) minimal medium. Glucose was used in these experiments instead of maltose, as several of the mutants were Mal− and displayed no growth in maltose-limited minimal medium. **(B)** Maximum growth rates in LB. Underlying data can be found in S1 Data. LB, Lysogeny broth; λ^VIR^, virulent mutant of phage λ.
(TIF)

S6 Fig Spot test assay for susceptibility to λ^VIR^. Plaque formation with a lawn bearing different fractions of λ^VIR^-susceptible and (*malT*^*−*^ *ompF*^*−*^) -resistant cells, 20 μL of a 5×10^9^ λ^VIR^ lysate spotted on to the lawn. When the frequency of susceptible cells is less than 0.01 (1%), there is no evidence for a reduction in the turbidity of the lawn. λ^VIR^, virulent mutant of phage λ.
(TIF)

S1 Table Number of colonies that were clearly Mal+ out of the five colonies tested at different transfers in the LB serial transfer experiment (Fig 2A). All colonies from both phage-free control cultures were susceptible to λ^VIR^. All of the tested colonies from the cultures with phage were resistant to λ^VIR^. Only colonies displaying confluent growth were counted as Mal+. LB, Lysogeny broth; Mal, maltose phenotype; λ^VIR^, virulent mutant of phage λ.
(XLSX)

S1 Data Numerical data used in figures. (XLSX)

## Abbreviations

cfu: colony-forming unit
CRISPR-Cas: Clustered regularly interspaced short palindromic repeats–CRISPR-associated proteins
IS: Insertion sequence
LB: Lysogeny broth
Mal+: phenotype characterized by the ability of bacteria to growth on maltose as a sole carbon source
M9M: M9 maltose medium
pfu: plaque-forming unit
wt: wild type
λ^KAN^: temperate (wild-type) phage λ generically marked with kanamycin resistance marker
λ^VIR^: virulent mutant of phage λ
