# Supplementary figures and images for: Leaky resistance and the conditions for the existence of lytic bacteriophage
Source: PLoS Biol. 2018 Aug 16;16(8):e2005971. doi: 10.1371/journal.pbio.2005971 (PMC6112682; doi:10.1371/journal.pbio.2005971)

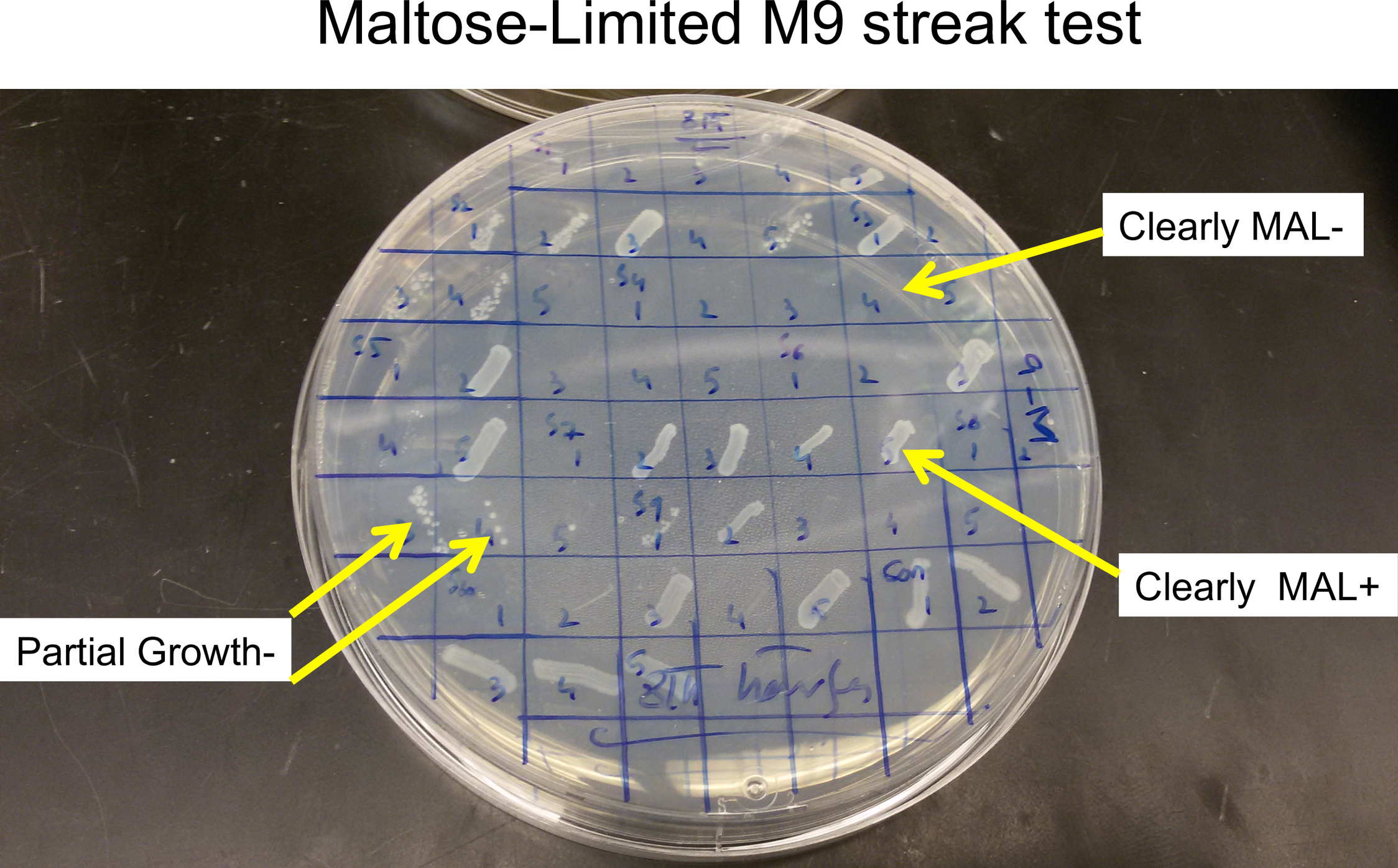

Supplement: S1 Fig — Both Mal+ and Mal− phenotypes are observed. Some isolates show partial growth and produces a few small colonies. Mal, maltose phenotype; λVIR, virulent mutant of phage λ. (TIF) [file pbio.2005971.s001.tif]

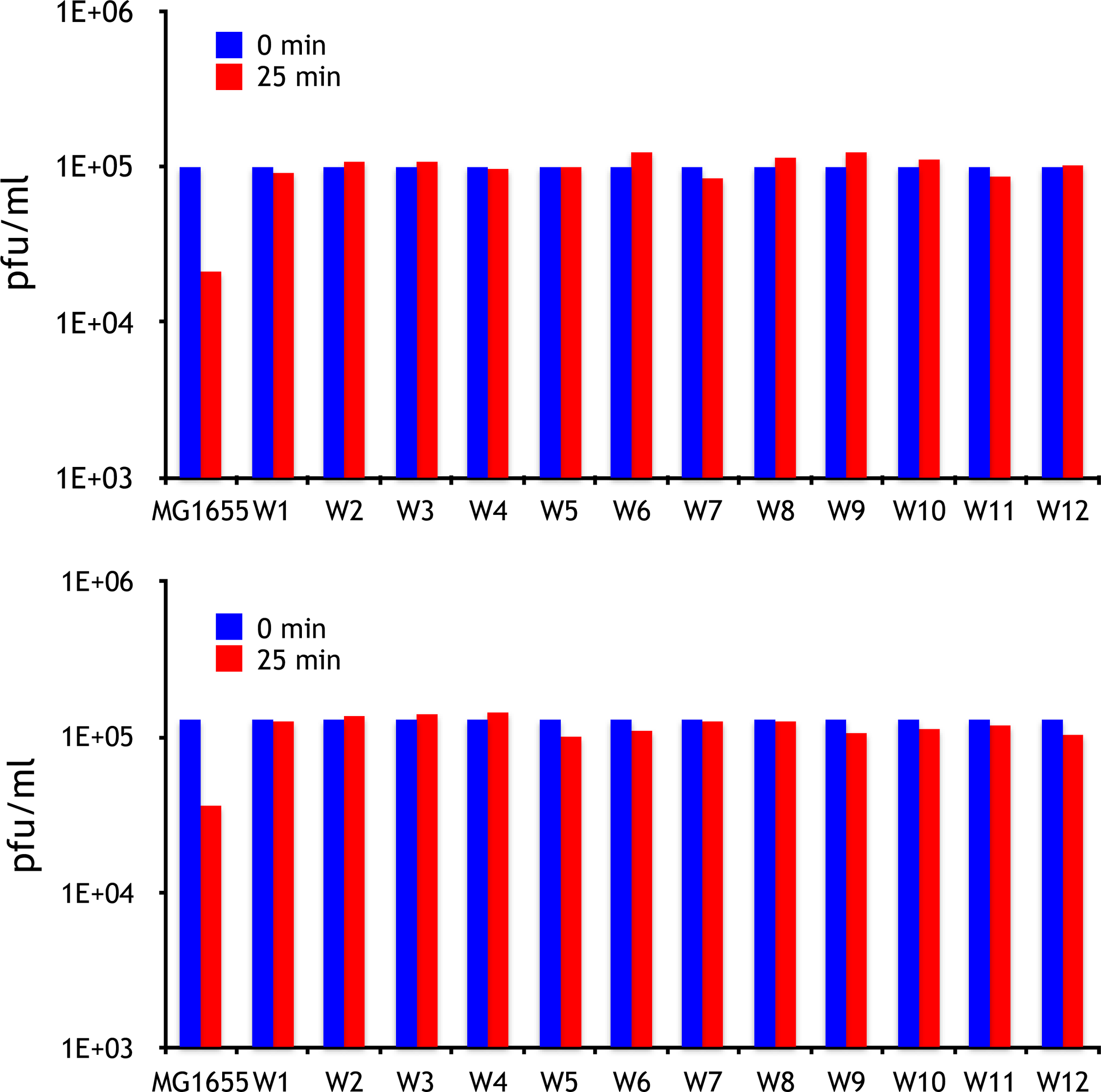

Supplement: S2 Fig — Densities of infective centers in two independent replicate experiments are shown. In each experiment, approximately 105 pfu/mL were mixed with 108 cfu/mL in 1 mL LB medium and the phage density was measured. After 25 minutes, the samples were chloroformed to kill all bacteria, including those that adsorbed phage, and the density of free phage was estimated by plating. In all samples, the bacterial density remained unchanged in the course of the experiment. Underlying data can be found in S1 Data. cfu, colony-forming unit; LB, Lysogeny broth; pfu, plaque-forming unit; λVIR, virulent mutant of phage λ. (TIF) [file pbio.2005971.s002.tif]

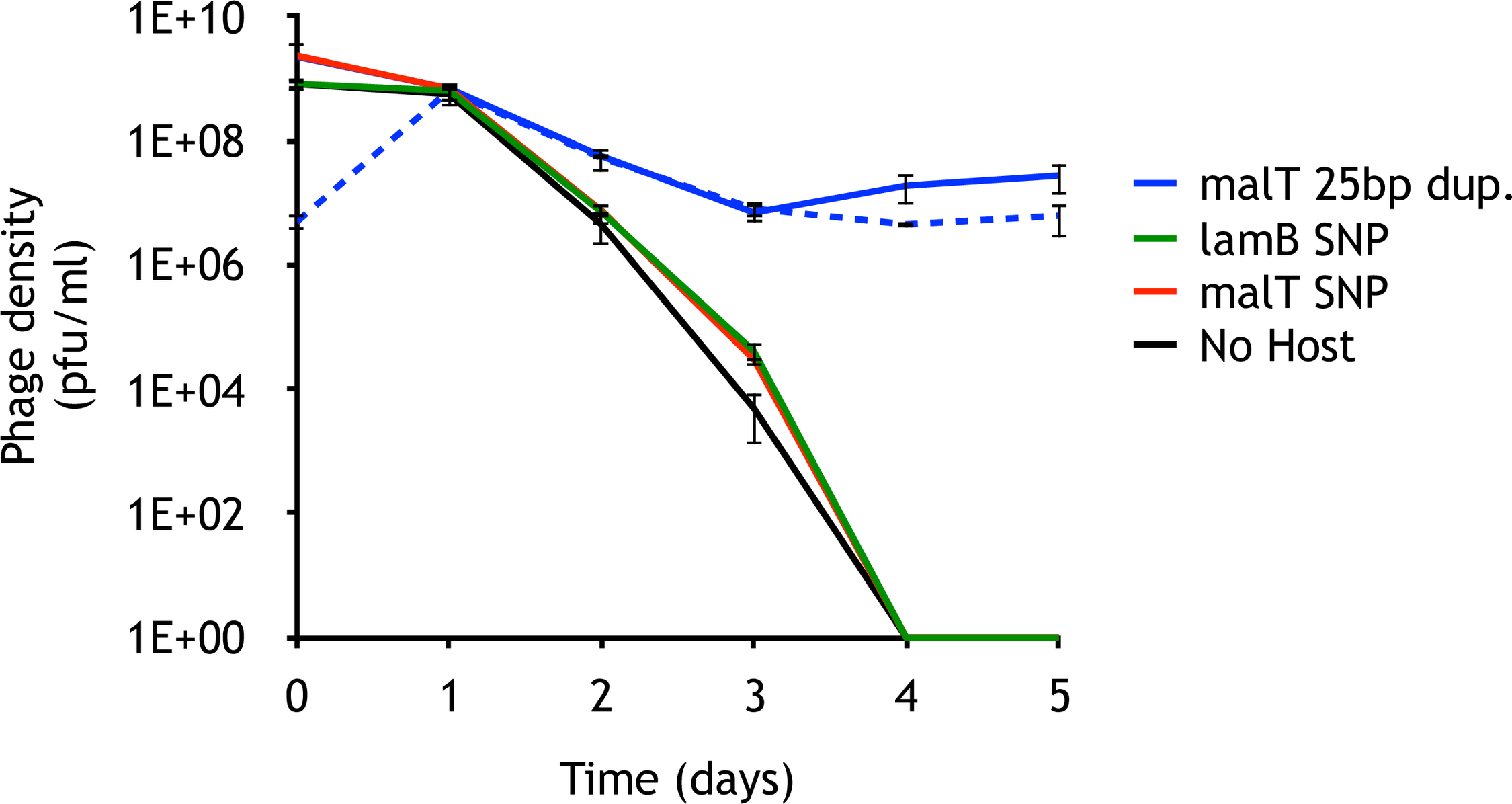

Supplement: S3 Fig — Serial transfer cultures were initiated with λVIR-resistant E. coli B (REL 606) mutants carrying either a 25-bp duplication in the malT gene, or independently isolated SNP mutations in malT (frameshift, due to a T inserted between nucleotides 610 and 611) and lamB (nonsense C->T at nucleotide 883 that creates a stop codon in AA #295) genes. The black line shows phage densities estimated in a serial transfer experiment with no host bacteria. Data points represent means of four biological replicates. Error bars represent the standard error of the mean (n = 4). The experiments were performed in M9 minimal medium supplemented with 1 mg/mL glucose as the limiting carbon source. Underlying data can be found in S1 Data. λVIR, virulent mutant of phage λ. (TIF) [file pbio.2005971.s003.tif]

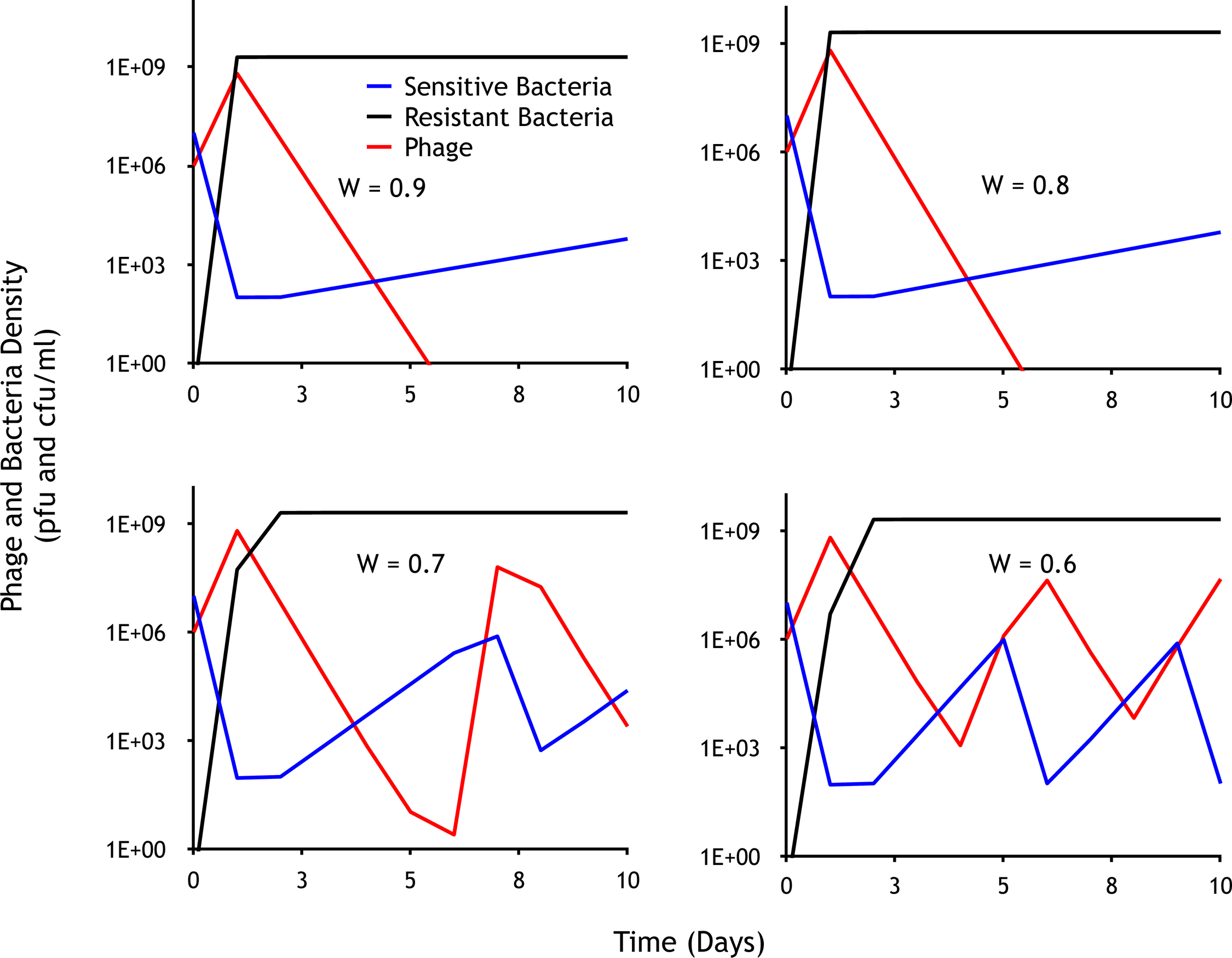

Supplement: S4 Fig — The parameter W, fitness, is the ratio of the maximum growth rate of the resistant clone, relative to the susceptible (vR/vS). In these simulations: δ = 2 × 10^−7^, β = 50, C = 1,000, v = 1, d = 0.01, e = 5 × 10^−7^, k = 1, μN = 5 × 10^−6^, μR = 0, ref = 102. In (A) and (B), the fitness cost of resistance is not sufficient to allow for phage maintenance. In (C) and (D), the phage is maintained as a result of the high fitness cost. Underlying data can be found in S1 Data. vR, maximum growth rate of resistant strain; vS, maximum growth rate of susceptible strain. (TIF) [file pbio.2005971.s004.tif]

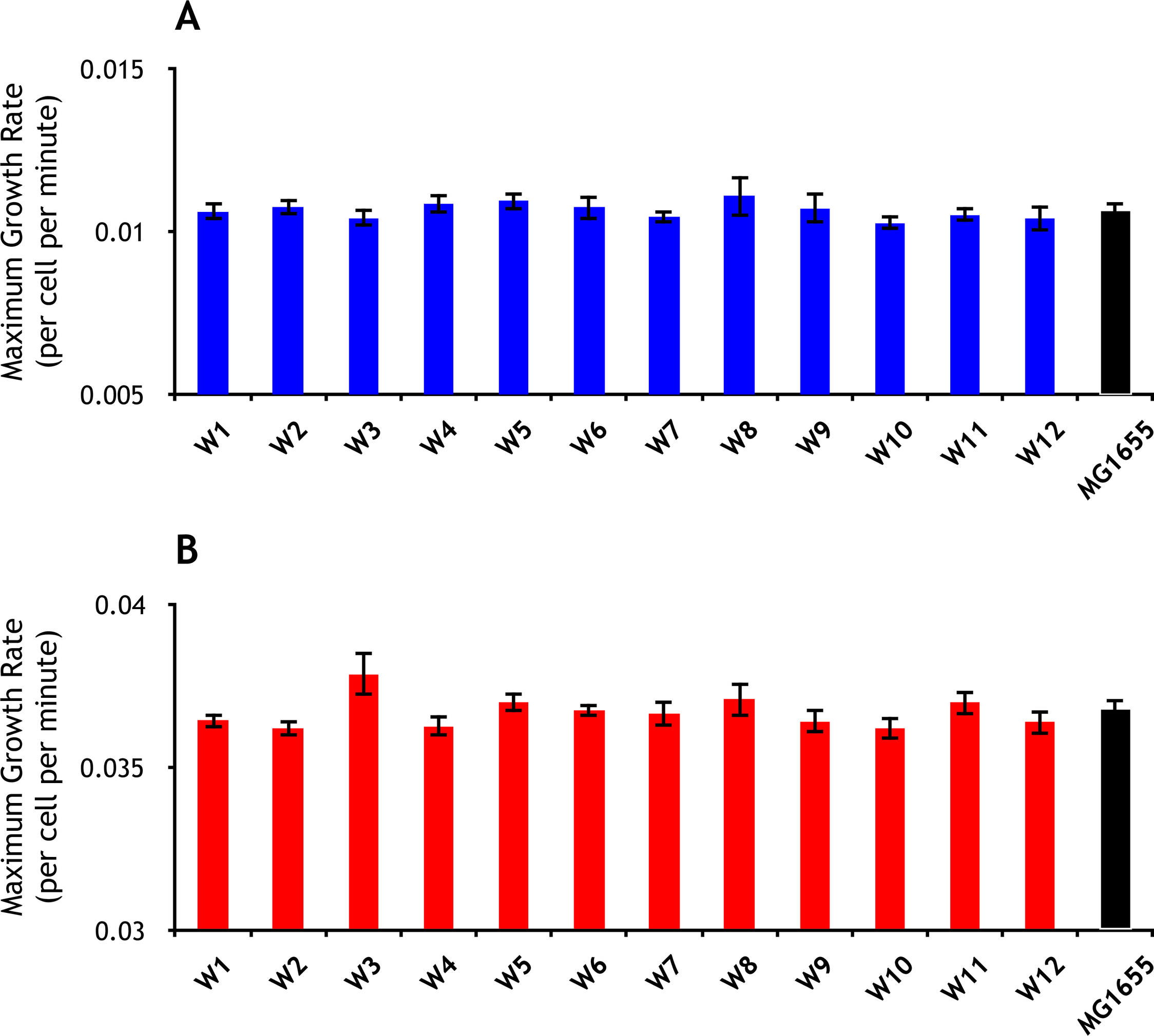

Supplement: S5 Fig — Mean and standard errors of five replicas are shown. The λVIR-susceptible ancestor strain is shown in black. (A) Maximum growth rates in M9 glucose (500 μg/mL) minimal medium. Glucose was used in these experiments instead of maltose, as several of the mutants were Mal− and displayed no growth in maltose-limited minimal medium. (B) Maximum growth rates in LB. Underlying data can be found in S1 Data. LB, Lysogeny broth; λVIR, virulent mutant of phage λ. (TIF) [file pbio.2005971.s005.tif]

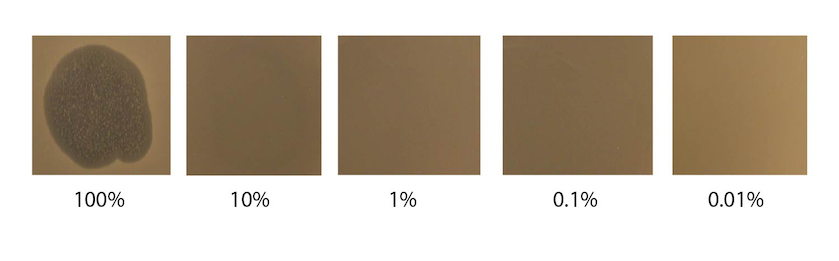

Supplement: S6 Fig — Plaque formation with a lawn bearing different fractions of λVIR-susceptible and (malT− ompF−) -resistant cells, 20 μL of a 5×10^9^ λVIR lysate spotted on to the lawn. When the frequency of susceptible cells is less than 0.01 (1%), there is no evidence for a reduction in the turbidity of the lawn. λVIR, virulent mutant of phage λ. (TIF) [file pbio.2005971.s006.tif]
